# Development and internal validation of a new life expectancy estimator for multimorbid older adults

**DOI:** 10.1186/s41512-025-00185-9

**Published:** 2025-03-04

**Authors:** Viktoria Gastens, Arnaud Chiolero, Martin Feller, Douglas C. Bauer, Nicolas Rodondi, Cinzia Del Giovane

**Affiliations:** 1 Institute of Primary Health Care (BIHAM), University of Bern, Bern, Switzerland; 2 Graduate School for Health Sciences, University of Bern, Bern, Switzerland; 3 Population Health Laboratory (#PopHealthLab), University of Fribourg, Fribourg, Switzerland; 4 School of Population and Global Health, McGill University, Montreal, Canada; 5 Department of General Internal Medicine, Inselspital, Bern University Hospital, University of Bern, Bern, Switzerland; 6 Departments of Medicine and Epidemiology & Biostatistics, University of California, San Francisco, CA USA

## Abstract

**Background:**

As populations are aging, the number of older patients with multiple chronic diseases demanding complex care increases. Although clinical guidelines recommend care to be personalized accounting for life expectancy, there are no tools to estimate life expectancy among multimorbid patients. Our objective was therefore to develop and internally validate a life expectancy estimator specifically for older multimorbid adults.

**Methods:**

We analyzed data from the OPERAM (OPtimising thERapy to prevent avoidable hospital admissions in multimorbid older people) study in Bern, Switzerland. Participants aged 70 years old or more with multimorbidity (3 or more chronic medical conditions) and polypharmacy (use of 5 drugs or more for > 30 days) were included. All-cause mortality was assessed during 3 years of follow-up. We built a 3-year mortality prognostic index and transformed this index into a life expectancy estimator. Mortality risk candidate predictors included demographic variables (age, sex), clinical characteristics (metastatic cancer, number of drugs, body mass index, weight loss), smoking, functional status variables (Barthel-Index, falls, nursing home residence), and hospitalization. We internally validated and optimism corrected the model using bootstrapping techniques. We transformed the mortality prognostic index into a life expectancy estimator using the Gompertz survival function.

**Results:**

Eight hundred five participants were included in the analysis. During 3 years of follow-up, 292 participants (36%) died. Age, metastatic cancer, number of drugs, lower body mass index, weight loss, number of hospitalizations, and lower Barthel-Index (functional impairment) were selected as predictors in the final multivariable model. Our model showed moderate discrimination with an optimism-corrected C statistic of 0.70. The optimism-corrected calibration slope was 0.96. The Gompertz-predicted mean life expectancy in our sample was 5.4 years (standard deviation 3.5 years). Categorization into three life expectancy groups led to visually good separation in Kaplan–Meier curves. We also developed a web application that calculates an individual’s life expectancy estimation.

**Conclusion:**

A life expectancy estimator for multimorbid older adults based on an internally validated 3-year mortality risk index was developed. Further validation of the score among various populations of multimorbid patients is needed before its implementation into practice.

**Trial registration:**

ClinicalTrials.gov NCT02986425. First submitted 21/10/2016. First posted 08/12/2016.

**Supplementary Information:**

The online version contains supplementary material available at 10.1186/s41512-025-00185-9.

## Introduction

Due to the aging of populations, there is a growing number of older patients with multiple chronic diseases requiring complex care. Providing high-value care among multimorbid older adults is however a major challenge due to polypharmacy, age-related physiological changes, and frailty. Evidence-based management of this type of patient is difficult also because they are frequently excluded from clinical trials [1].

Because multimorbid patients have a relatively short life expectancy (LE), many guidelines recommend personalized care accounting for LE. For instance, the European Society of Cardiology recommends that “life expectancy has to be taken into account when starting a new medication” [2]. The American College of Cardiology and American Heath Association recommends “for older adults with […] limited life expectancy, clinical judgment, patient preference, and a team-based approach” [3]. This is especially important for preventive care because there is a lag time to benefit. For instance, the lag time to benefit from breast cancer screening has been estimated to be 7 years [4, 5]. As older multimorbid patients have a relatively short LE, they might not have the time to benefit.

There is however no tool to accurately estimate LE among multimorbid patients. LE estimations are usually derived from life tables [6]. Life tables are built from general population data and are therefore of limited use for specific patient groups as they generally only differentiate by age and sex. Common mortality prognostic factors such as clinical characteristics and functional limitations are generally not considered in life tables. To mitigate this shortcoming, one may derive LE estimations from mortality prediction indices as they incorporate multiple patient-level risk factors [7, 8]. To our knowledge, only Lee et al. have developed a LE estimator from a mortality risk prognostic index from a cohort of community-dwelling older adults with a relatively low mortality rate [7]. It is however not directly applicable to multimorbid patients and other populations with relatively high mortality rates.

Our objective was therefore to develop and internally validate for the first time a LE estimator for older multimorbid adults.

## Methods and analysis

We first built and validated internally a 3-year mortality prognostic index in a cohort of multimorbid older adults and, second, transformed this mortality index into an LE estimator. The protocol of this study and previous results for a 1-year mortality prognostic index have been published previously [8, 9].

### Source of data and study design

We used data from 822 participants of an ongoing cohort study in Bern, Switzerland. Participants were originally enrolled in the clinical trial OPtimising thERapy to prevent Avoidable hospital admissions in Multimorbid older people (OPERAM [8, 10]). This trial was first posted on ClinicalTrials.gov on 08/12/2016 (NCT02986425). Participants were assigned to receive either standard care or a medication review by a Systematic Tool to Reduce Inappropriate Prescribing (STRIP) with observation of the primary outcome of drug-related hospital admission (DRA) over 1 year.

For the current study, we used data collected at baseline (December 2016–October 2018) and up to 3 years after baseline (until October 2021). The local Ethics Committee (“Kantonale Ethikkommission Bern”) in Bern, Switzerland, approved the study protocol with the project number 2018–00784. Study nurses collected baseline data through a personal interview with the participant and from medical files. The follow-up was conducted via phone calls. Phone interviews were held with participants or relatives, otherwise with a proxy or with the general practitioner, when the participants were not reachable or not able to answer.

We developed and validated the mortality prognostic index following the Prognosis Research Strategy (PROGRESS) framework [11] and reported it following the Transparent Reporting of a multivariable prediction model for Individual Prognosis Or Diagnosis (TRIPOD) statement [12, 13]. We further followed the recommendations of Moons et al. [14, 15] for risk prediction models. This study is part of a research project whose protocol has been published previously [8].

### Participants

Participants (*N* = 822) were enrolled at the time of hospitalization in the Inselspital, University Hospital, Bern, Switzerland. Inclusion criteria were the age of 70 years or older, multimorbidity (3 or more chronic medical conditions), and polypharmacy (use of 5 drugs or more for > 30 days) [8, 9].

Written informed consent by patients themselves or, in the case of cognitive impairment by a legal representative, had already been obtained before enrolment. Patients planned for direct admission to palliative care (< 24 h after admission), patients undergoing a structured drug review other than the trial intervention, or those who had passed a structured drug review within the last 2 months were excluded. Patients for whom it was not possible to obtain informed consent were excluded [8, 9].

### Outcome

The primary outcome was time to all-cause mortality over 3 years of follow-up. Information on death and relative dates was collected by study nurses through follow-up calls or primary care physician contact.

### Candidate 3-year mortality predictors

To build the mortality index, we identified potential mortality predictors. Candidate 3-year mortality predictors were derived from previous research efforts in this field [16, 17], ease and reliability of measurement in clinical settings, and background knowledge on potential associations with mortality. We also considered factors included in the OPERAM dataset that may not be identified from the literature but are specific to multimorbid patients. All candidate predictors were based on characteristics measured at inclusion in the cohort. We included demographic variables (age, sex), clinical characteristics (number of drugs, body mass index, weight loss during the last year, metastatic solid tumor), smoking, functional status variables (Barthel-Index, falls, nursing home residence), and hospitalization. We identified in a previous study the diagnosis of a metastatic solid tumor as the strongest predictor variable of the Charlson-Comorbidity-Index for 1-year mortality in older multimorbid adults [9]. The variables about falls and hospitalization reflected the number of events during the last 12 months before index hospitalization. The Barthel-Index measures performance in activities of daily living (ADL) on an ordinal scale from 0 to 100 with higher scores indicating more independence [18]. Continuous candidate predictors were analyzed as continuous variables and not categorized.

### Sample size

We have calculated the required sample size for conducting our multivariable prediction model utilizing the criteria proposed by Riley et al. [19] and implemented in the *pmsampsize* library for the R environment [20]. The minimum sample size required with 12 candidate predictor parameters, an expected outcome event rate of 0.1 per year, and an anticipated Cox-Snell R2 of 0.126 (C statistic of [17] 0.82 [19]) is 799 with 20 events per predictor parameter. This is considerably more than the idiomatic 10 events per predictor parameter. Our sample size of 805 is therefore adequate for this project.

### Missing data

We used multiple imputations (number of multiple imputations, *m* = 5) for missing values under a missing at-random assumption in order to reduce bias and avoid excluding participants from the analysis [14, 21]. We used the *mice* library for multiple imputation and pooling in the R environment [22].

### Statistical analysis

We applied a parametric Weibull regression with the least absolute shrinkage and selection operator (LASSO) penalization to perform predictor selection. The Weibull hazard function is given by $h\left(t\right)=\lambda \alpha {t}^{\alpha -1}$ with the scale parameter λ and the shape parameter α, respectively. We have obtained the LASSO regularization parameter lambda using cross-validation with the R function *cv.glmnet* (lambda = 0.032). The final model was fitted in each imputed dataset and results were pooled according to Rubin’s rules. We investigated the predictive accuracy of the final model by testing calibration and discrimination. The apparent performance and discrimination of the model were assessed with C statistic [14]. We evaluated potential overfitting and optimism by internal validation with bootstrapping techniques [14, 15]. We performed 500 bootstrap cycles. In each bootstrap sample, we derived a mortality prediction model and the relative risk score, as done in the original sample. We calculated optimism as the difference in performance measure (C statistic) between the original sample and the respective bootstrap sample. This was repeated for all bootstrap samples to estimate the average optimism. We evaluated the calibration slope and intercept (calibration-in-the-large).

We transformed the 3-year prognostic index into an LE estimator following the method of Lee et al. [7] In particular, we used the new 3-year mortality prognostic index to define subpopulations with the same risk score. We calculated the risk score based on the β coefficients (logHR) of the final model. We fitted a Gompertz survival function with each risk score as a predictor having a flexible proportional effect on the hazard rate. The Gompertz function assumes that each subpopulation will experience an exponential rise in mortality risk over time (*h*_*i*_(*t*) = *λ*_*i*_ exp(*γt*), where *λ*_*i*_ = exp(*x*_*i*_*β*)). We compared our fitted Gompertz model with observed Kaplan–Meier survival curves in three equally sized risk groups. We built an interactive web application of the final model using the *Shiny* package in the R environment [23].

## Results

Of the 822 participants in the cohort, 805 were included in the analytical sample. We excluded 17 participants because they left the study and most data were missing. Baseline characteristics of the participants are reported in Table 1. The mean (standard deviation, min to max) age of participants was 79.7 (6.5, 70 to 99) years and 42% were women. During 36 months of follow-up, 292 participants (36%) had died. Missing data of the candidate predictors was between 0% and 3.7% (Table S1). Given the low occurrence of missing data and the use of multiple imputations, we did not perform sensitivity analysis regarding missing data.

**Table 1:** Baseline characteristics of all participants and of those who died during follow-up

**Variables**		**Total** ***n* = 805**^**a**^	**Deaths** ***n* = 292**
Age	Mean (SD^b^) [min; max]	79.7 (6.5) [70; 99]	81.5 (7.1) [70; 99]
Sex	Female, *n* (%)	338 (42)	125 (43)
	Male, *n* (%)	467 (58)	167 (57)
Metastatic solid tumor	Yes, *n* (%)	49 (6)	32 (11)
	No, *n* (%)	756 (94)	260 (89)
Number of drugs^c^	Mean (sd) [min; max]	11.2 (5.1) [5; 38]	12.3 (5.7) [5; 38]
Body mass index (BMI)	Mean (sd) [min; max]	26.7 (5.3) [13.3; 56.7]	25.7 (5.3) [13.3; 50.9]
Weight loss^d^	Yes, *n* (%)	255 (32)	107 (37)
	No, *n* (%)	545 (68)	183 (63)
Current smoker	Yes, *n* (%)	69 (9)	25 (9)
	No, *n* (%)	733 (91)	266 (91)
Hospitalizations^d^	Mean (sd) [min; max]	1.1 (1.6) [0; 20]	1.3 (1.7) [0; 10]
Barthel-Index	Mean (sd) [min; max]	80.0 (25.3) [0; 100]	71.7 (29.1) [0; 100]
Falls^d^	Mean (sd) [min; max]	2.6 (30.3) [0; 800]	2.4 (17.8) [0; 300]
Nursing home residency	Yes, *n* (%)	70 (9)	39 (13)
	No, *n* (%)	735 (91)	253 (87)

^a^Participants with lost to follow-up (*n* = 37) or withdrawal (*n* = 30) were included until the last day of contact, participants not consenting to the 3-year follow-up were included until the 1-year follow-up (*n* = 72)

^b^Standard deviation

^c^Before index hospitalization

^d^During the last 12 months

**Fig. 1:**
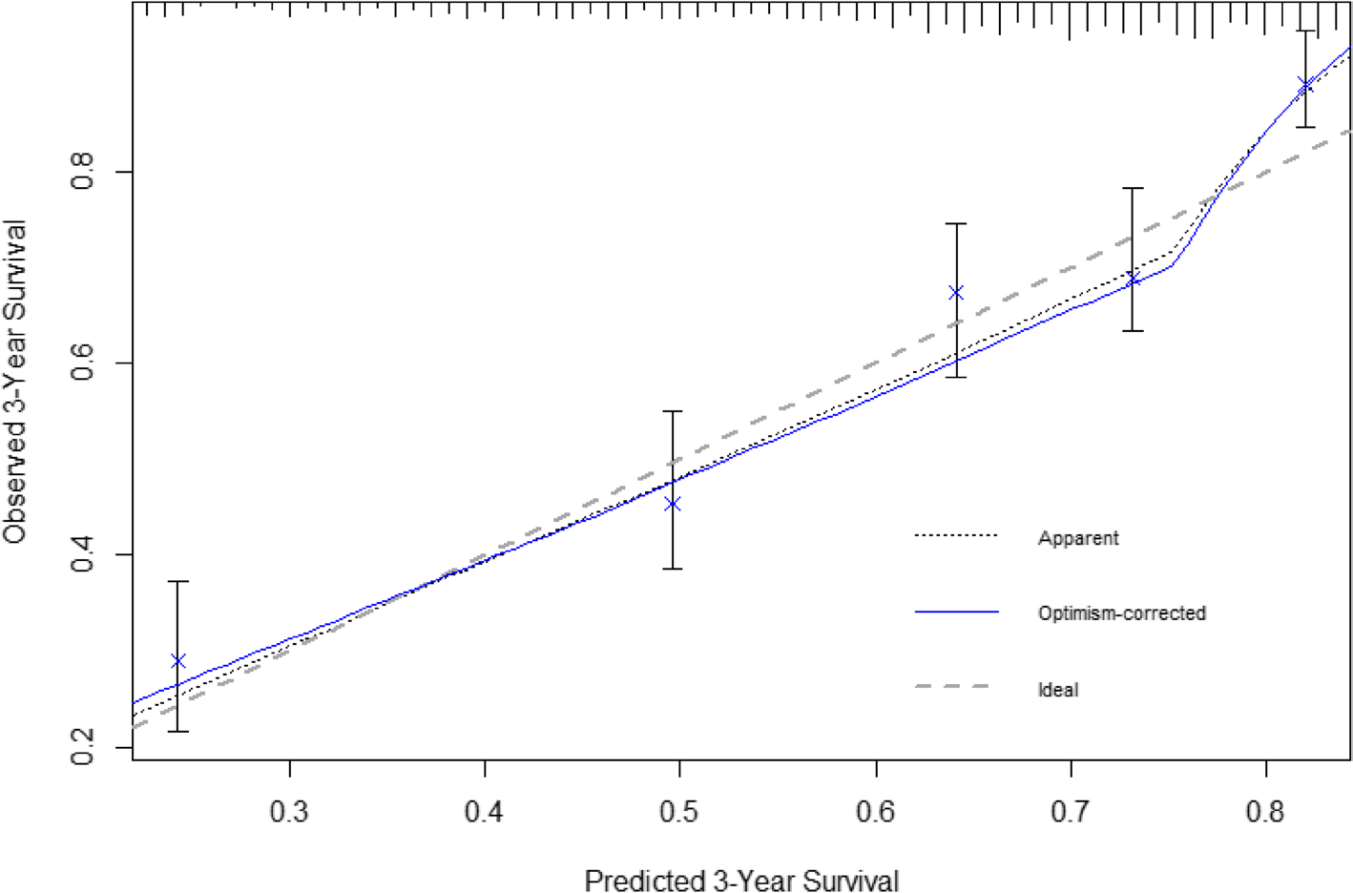
Calibration plot of the prediction model for 3-year mortality with the apparent (dotted black) and optimism-corrected (blue) lines

The final 3-year mortality risk prediction model included age, BMI, metastatic solid tumor, hospitalizations, drugs, Barthel-Index, and weight loss as predictors (Table 2). The number of events per predictor (also called events per variable; EPV) was 26.5. We calculated the risk score based on the β coefficients of the final model (Table 2), where $\textit{risk score}=0.05\;\textit{age}+1.24\;\textit{metastatic solid tumour}+0.05\;n\left(\text{drugs}\right)-0.04\;\textit{BMI}+0.25\;\textit{weight loss}+0.05\;n\left(\text{hospitalizations}\right)-0.0\;5\;\textit{Barthel}-\textit{index}$. The variables *metastatic solid tumour* and *weight loss* were binary variables [no 0; yes 1]. Table 3 shows apparent and internal validation performance statistics of the 3-year mortality risk prediction model. After adjustment for optimism with bootstrapping, our final model was able to discriminate participants with and without death within 3 years with a C statistic of 0.70. The optimism-corrected calibration slope was 0.96. Figure 1 shows the calibration plot of the mortality risk prediction model. The optimism-corrected model performs slightly worse than the apparent model, e.g., indicating slightly higher underprediction at low (< 0.3) predicted probabilities.

**Table 2:** 3-year mortality predictors retained in the final Weibull model (scale parameter λ = 0.0001, shape parameter α = 0.81) and mortality risk associated (*HR* hazard ratio, *ETR* event time ratio, *CI* confidence interval)

**Variable**		**HR (95% CI)**	**β coefficient**^**a**^	**ETR (95% CI)**	***p*-value**
Age (year)		1.06 (1.04–1.07)	0.05	0.94 (0.92–0.96)	< .001
Metastatic solid tumor	No	Ref	Ref	Ref	
	Yes	3.46 (2.42–4.95)	1.24	0.20 (0.13–0.32)	< .001
Number of drugs		1.05 (1.03–1.07)	0.05	0.94 (0.92–0.97)	< .001
Body mass index (kg/m^2^)		0.96 (0.94–0.99)	− 0.04	1.04 (1.02–1.07)	< .01
Weight loss	No	Ref	Ref	Ref	
	Yes	1.29 (1.02–1.63)	0.25	0.73 (0.55–0.98)	0.04
Hospitalizations (number during last year)		1.05 (1.00–1.11)	0.05	0.93 (0.88–0.99)	0.04
Barthel-Index (score for activities of daily living)		0.99 (0.98–0.99)	− 0.01	1.02 (1.01–1.02)	< .001

^a^β coefficient = logHR

**Table 3:** Apparent and internal validation performance statistics of the final prediction model (with 95% CI) including C statistic, calibration slope, and calibration-in-the-large

**Performance measure**	**Apparent**	**Average optimism**	**Optimism corrected**
C statistic	0.72	0.02	0.70 (0.69–0.70)
C slope	1	0.04	0.96 (0.95–0.96)
CITL	0	− 0.32	0.32 (0.31–0.33)

**Fig. 2:**
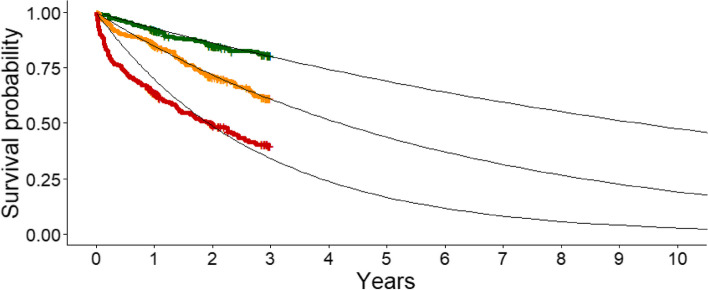
Observed Kaplan–Meier survival curves of the three life expectancy risk groups (high risk (red), intermediate risk (orange), low risk (green)) combined with their Gompertz-model predicted life expectancy estimations over 10 years (black)

The Gompertz-predicted mean LE was 5.4 years (standard deviation 3.5), and it ranged from 0.2 to 21.7 years. We created three equally sized risk groups based on the derived point-based risk score (Table 4). Figure 2 shows the Kaplan–Meier plot of the three LE groups and reveals a good graphical discrimination.

**Table 4:** Risks groups defined by prognostic index points and their estimated life expectancy

**Risk group**	**Prognostic index**	**Gompertz-predicted mean life expectancy (95% CI), in years**
Low	− 2.5 to − 1.1	9.0 (8.7–9.3)
Intermediate	− 1.0 to − 0.1	4.5 (4.3–4.7)
High	0.0 to 4.7	2.4 (2.2–2.6)

In a sensitivity analysis, we applied the Weibull model also for the life expectancy extrapolation (instead of the Gompertz model). The corresponding Kaplan–Meier plot is displayed in Fig. S1 and shows an improved graphical discrimination.

We have also developed a web application where personalized life expectancy estimations are calculated automatically. This is available for use in a R Shiny app (https://vgastens.shinyapps.io/leshiny/).

## Discussion

We developed a prognostic model that estimates LE for multimorbid older individuals. Our model included seven prognostic factors: age, metastatic solid tumor, number of drugs, body mass index, weight loss, number of hospitalizations, and Barthel index. Our model showed moderate discrimination.

Prognostication is essential for risk perception and informed decision-making. Until now, no widely accepted prognostic model for LE in multimorbid patients has been used in clinical practice. Our recent external validation of six mortality risk scores concluded that “the search for a more accurate mortality score remains” [24]. This is in line with Yourman et al. concluding in their systematic review of mortality indices for older adults that none could be recommended for widespread use [15].

In comparison to previously published prognostic models, we argue that the model we present in this study offers several advantages: firstly, our study adheres to reporting guidelines and research frameworks of the field [11, 13]. This includes the internal validation we performed to account for optimism. Secondly, we performed multiple imputations for missing data to minimize potential bias induced, avoided categorization, and kept variables continuous [21]. Thirdly, we developed a web-based application where doctors and patients can potentially enter the patient-level risk factors easily (https://vgastens.shinyapps.io/leshiny/), estimate LE, and take it into account for informed decision-making. This tool shows the potential of the proposed approach, however, it is not yet ready for use in clinical practice, as decision-making tools need external validation with an independent cohort of patients.

The applicability of our model is limited by several factors beyond the needed external validation. We included hospitalized individuals. They showed an expected higher mortality closer to the index hospitalization. Hospitalized individuals might have a higher morbidity and mortality than non-hospitalized individuals. The available follow-up data was limited to 3 years, extrapolation beyond contains a higher level of uncertainty. In addition, the sample size of the OPERAM cohort is relatively small compared to other observational studies; this study though is of high quality. In general, the LE estimation should support clinical judgment rather than replace it. Single-number survival predictions for individuals may provide a false sense of precision given the uncertainty in predictions for patients (as opposed to populations) [7, 25]. Since the sensitivity analysis showed improved performance in the pure Weibull model, we would consider pursuing this methodology for the next projects.

We developed and internally validated the first LE estimator specifically for multimorbid adults. As the next step, the model should be externally validated in an independent dataset. Our web application, when externally validated, could be used by patients and doctors to estimate LE and to support informed decision-making.

## Supplementary Information

Supplementary Material 1: Table S1. Missing data in the candidate predictors of the 805 participants. Figure S1. Observed Kaplan-Meier survival curves of the three life expectancy risk groups (high risk (red), intermediate risk (orange), low risk (green)) combined with their Weibull-model predicted life expectancy estimations over ten years (black).

## Data Availability

This study involves human research participant data containing sensitive patient information. In the EU Horizon 2020 grant agreement for the OPERAM study, it had been specified that the data would be made available upon request if the use had been approved by an ethical committee. Therefore, restrictions to make the underlying data directly publicly available are both due to legal and ethical reasons, as health data are sensitive data. Data for this study will be made available for scientific purposes upon request for researchers whose proposed use of the data has been approved by the OPERAM publication committee. After approval and signing of a data transfer agreement ensuring adherence to privacy and data handling, data and documentation will be made available through a secure file exchange platform. Partially de-identified participant data, a data dictionary, and annotated case report forms will be made available. For data access, external researchers can visit www.operam-cohort.biham.ch and email operam[at]biham.unibe.ch.
