# Supplementary material for: Development and internal validation of a new life expectancy estimator for multimorbid older adults
Source: Diagn Progn Res. 2025 Mar 4;9:5. doi: 10.1186/s41512-025-00185-9 (PMC11877760; doi:10.1186/s41512-025-00185-9)

**SUPPLEMENTAL MATERIAL**

Development and internal validation of a new life expectancy estimator for multimorbid older adults

Viktoria Gastens, Arnaud Chiolero, Martin Feller, Douglas C. Bauer, Nicolas Rodondi, and Cinzia Del Giovane

**Table S1.** Missing data in the candidate predictors of the 805 participants.

| Variable | Missing data, n (%) |
| --- | --- |
| Age | 0 |
| Sex | 0 |
| Metastatic solid tumor | 0 |
| Number of drugs | 0 |
| Body mass index | 30 (3.7) |
| Weight loss | 5 (0.6) |
| Current smoker | 3 (0.4) |
| Hospitalisations | 3 (0.4) |
| Barthel-Index | 13 (1.6) |
| Falls | 4 (0.5) |
| Nursing home residency | 0 |

**Figure S1.** Observed Kaplan-Meier survival curves of the three life expectancy risk groups (high risk (red), intermediate risk (orange), low risk (green)) combined with their Weibull-model predicted life expectancy estimations over ten years (black).
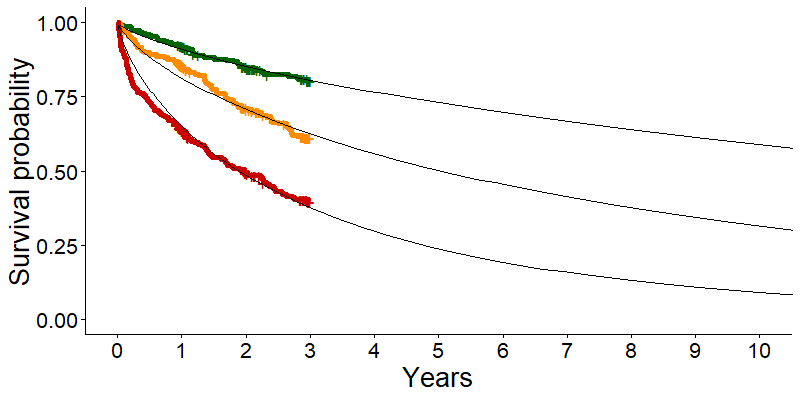

Supplement: Supplementary file 1 — Supplementary Material 1: Table S1. Missing data in the candidate predictors of the 805 participants. Figure S1. Observed Kaplan-Meier survival curves of the three life expectancy risk groups (high risk (red), intermediate risk (orange), low risk (green)) combined with their Weibull-model predicted life expectancy estimations over ten years (black). [file 41512_2025_185_MOESM1_ESM.docx]
